# Structure of human NCC: insights into the inhibition mechanism of thiazides

**DOI:** 10.1038/s41392-023-01527-z

**Published:** 2023-07-03

**Authors:** Livia de Souza Goncalves, Pattareeya Yottasan, Onur Cil

**Affiliations:** Department of Pediatrics, University of California, San Francisco, San Francisco, CA USA

**Keywords:** Structural biology, Nephrology

A recent study published in *Nature* by Fan et al.^1^ identified the structure of human NCC alone and in complex with a thiazide diuretic using cryo-electron microscopy. Despite extensive clinical use of NCC inhibitor thiazide diuretics since 1950s, the structure and inhibition mechanisms of NCC have remained elusive, and Fan et al. study is a major step toward better understanding the NCC biology and mechanism of action of thiazide diuretics.

**Fig. 1:**
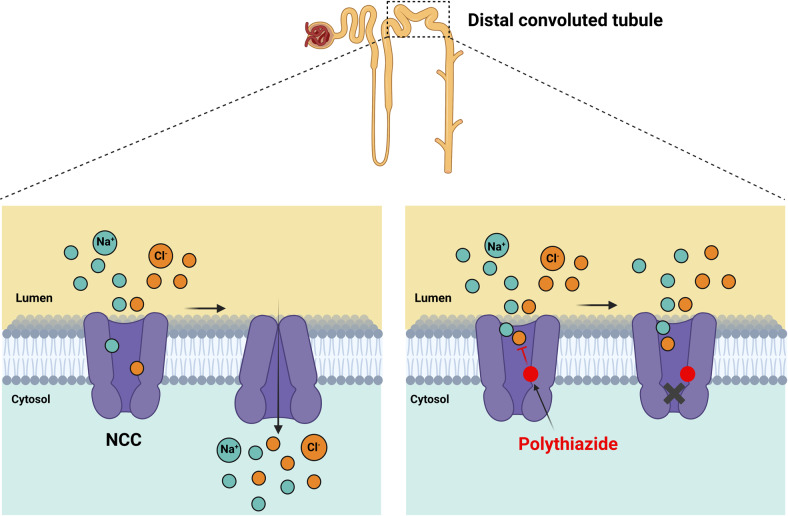
Proposed molecular mechanisms of NCC inhibition by thiazide diuretics. Polythiazide competes with Cl^−^ for the same binding site and prevents conformational change of the transporter. Created in BioRender.com

Sodium-chloride cotransporter (NCC, also known as SLC12A3) is expressed in the kidney where it facilitates electroneutral NaCl absorption. NCC belongs to the cation-chloride cotransporters (CCCs) family, which transport Cl^−^ together with Na^+^ and/or K^+^, and play critical roles in several physiological processes. Other members of CCC family include NKCCs and KCCs, for which recent structural biology studies provided key insights, however the detailed structure of NCC remained unknown. Fan et al.^1^ confirmed that NCC is a dimer and each subunit consists of transmembrane domain (transport unit) and cytosolic regulatory N-terminal and C-terminal domains (NTD and CTD, respectively). They found that the Na^+^ and Cl^−^ binding sites of NCC are conserved with NKCCs and KCCs; however, the critical K^+^-coordinating tyrosine is replaced with histidine (H234) in NCC that potentially explains why it cannot transport K^+^. From structural and functional studies, they identified that polythiazide (a thiazide diuretic) inhibits NCC mainly by two mechanisms: (1) it competes with the Cl^−^ for the same binding site; (2) it prevents isomerization of the NCC from the outward-facing to inward-facing conformation and inhibits NCC’s transport cycle (Fig. 1). NCC activity is mainly regulated by phosphorylation along its NTD by serine-threonine kinases SPAK (STE20/SPS1-related proline/alanine-rich kinase) and OSR1 (oxidative stress-responsive kinase 1). These kinases are activated by upstream enzymes including SGK1 (glucocorticoid regulated kinase 1) and WNKs (with no lysine kinases) in response to distinct mediators such as aldosterone and angiotensin II.^2^ Upon activation by SGK1 and WNKs, SPAK and OSR1 can phosphorylate serine and threonine residues in the cytoplasmic domain of the NCC, which allow NaCl absorption from the lumen of the distal convoluted tubule. Fan et al.^1^ showed that critical residues for phosphorylation are located at the interaction region of the regulatory cytosolic NTD and CTD. Since phosphorylation at the NTD of the NCC stimulates its transport activity, these new data suggest that phosphorylation may regulate the NCC function by modulation of NTD-CTD interaction and/or inducing conformational shift. Together, these findings reveal key insights for understanding the mechanisms of NaCl transport by NCC and its inhibition by thiazide diuretics.

NCC is expressed in the apical membrane of the distal convoluted tubule (DCT) in the kidney. It mediates reabsorption of 5–10% of the filtered NaCl and regulates electrolyte and acid-base balance. Loss of function mutations in *SLC12A3* (encoding NCC), result in autosomal recessive Gitelman syndrome characterized with hypokalemia, hypomagnesemia, metabolic alkalosis and hypercalciuria which resembles the effects of chronic thiazide diuretic use. However, whether thiazides only inhibit NCC for urinary effects of, or also inhibit other transporters such as Na^+^-dependent Cl^−^/HCO_3_ exchanger (NCDBE or SLC4A8) remains controversial.^3^ Nevertheless, Fan et al.^1^ study also provides critical insights regarding the structural and functional effects of certain Gitelman syndrome mutations.

Despite many newer classes of antihypertensives being available, thiazides remain one of the most commonly used first-line agents to treat essential hypertension. In addition to monotherapy, thiazides are commonly used in combination with other drugs in hypertension.^4^ However, the exact mechanisms of antihypertensive action of thiazides remain controversial. Thiazides are weaker diuretics compared with loop diuretics; however, they have greater blood pressure lowering effect, which suggests additional mechanisms for their antihypertensive action. Various mechanisms other than NCC inhibition and diuresis have been proposed for the antihypertensive effects of thiazides including reduction of peripheral resistance by direct and indirect effects.^4^ Regardless of the mechanisms of their antihypertensive effects, an important issue with thiazide use is the metabolic side effects (hyperglycemia, hyperlipidemia and hyperuricemia). The exact mechanisms of these metabolic abnormalities remain unclear; however, they have not been consistently reported in Gitelman syndrome patients, which implicates potential off-target effects of thiazides as the culprit. This is also suggested by a recent mouse study showing that thiazides inhibit mitochondrial carbonic anhydrase isoform 5b which results in attenuated insulin secretion in pancreatic β-cells.^5^ Although Fan et al. study^1^ is an important step toward understanding the mechanism of action of thiazides, future studies are required to elucidate exact mechanisms for their metabolic side effects, which can ultimately enable development of thiazide or thiazide-like compounds with diuretic and antihypertensive effects but minimal metabolic side effects.
